# Supplementary material for: Identification of photoperiod-induced specific miRNAs in the adrenal glands of Sunite sheep (Ovis aries)
Source: Front Vet Sci. 2022 Jul 22;9:888207. doi: 10.3389/fvets.2022.888207 (PMC9354845; doi:10.3389/fvets.2022.888207)
Supplement: Supplementary file 1 [file Data_Sheet_1.ZIP › Supplementary file/miRDeep2 file.zip!/miRDeep2 file/Know_Structure/LP42A1/oar-mir-181a-1.pdf]

miRBase precursor : oar-mir-181a-1
Total read count : 23199
oar-miR-181a read count : 23087
remaining reads : 112

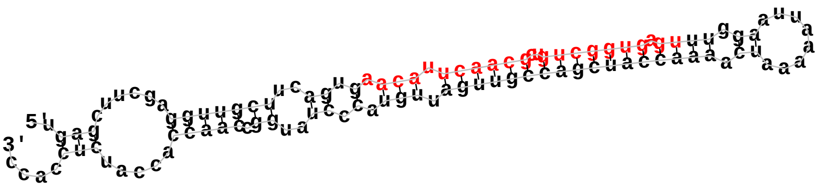

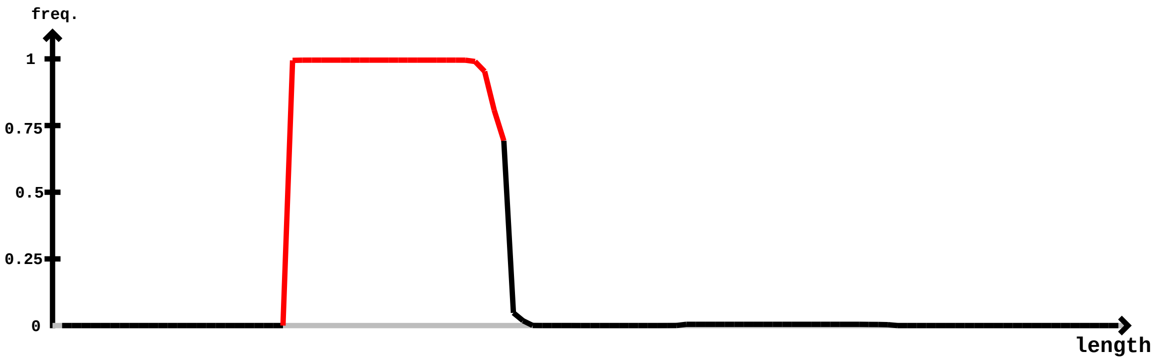


```
                          oar-miR-181a
5'- ugagcuucgagguugcuucagugaacauucaacgcugucggugaguuuggaauuaaaaaucaaaaccaucgaccguugauuguacccuauggccaaccaccaucuccacc   -3'    exp
    .(((......(((((((..((.(.(((.((((((..(((((((.((((.((........)).))))))))))))))))).))).).))..)).))))).....)))....   reads     mm      sample
    .......................aacauucaacgcugucgg.....................................................................   4         0       seq
    .......................aacauucaacgcugucggu....................................................................   115       0       seq
    .......................aacauucaacgcugucggug...................................................................   858       0       seq
    .......................aacauucaacgcugucgguga..................................................................   3381      0       seq
    .......................aacauucaacgcugucggugag.................................................................   2653      0       seq
    .......................aacauucaacgcugucggugagu................................................................   14955     0       seq
    .......................aacauucaacgcugucggugaguu...............................................................   682       0       seq
    .......................aacauucaacgcugucggugaguuu..............................................................   412       0       seq
    .......................aacauucaacgcugucggugaguuug.............................................................   6         0       seq
    .......................aacauucaacgcugucggugaguuuggaau.........................................................   2         0       seq
    .......................aacauucaacgcugucggugaguuuggaauu........................................................   1         0       seq
    ........................acauucaacgcugucggug...................................................................   2         0       seq
    ........................acauucaacgcugucgguga..................................................................   1         0       seq
    ........................acauucaacgcugucggugag.................................................................   2         0       seq
    ........................acauucaacgcugucggugagu................................................................   11        0       seq
    ........................acauucaacgcugucggugaguu...............................................................   1         0       seq
    ........................acauucaacgcugucggugaguuu..............................................................   1         0       seq
    .........................cauucaacgcugucggugagu................................................................   1         0       seq
    .........................cauucaacgcugucggugaguuu..............................................................   1         0       seq
    ...........................uucaacgcugucggugagu................................................................   1         0       seq
    ..............................................................aaaccaucgaccguugauugu...........................   2         0       seq
    ...............................................................aaccaucgaccguugauugu...........................   1         0       seq
    ...............................................................aaccaucgaccguugauugua..........................   2         0       seq
    ...............................................................aaccaucgaccguugauuguac.........................   1         0       seq
    ...............................................................aaccaucgaccguugauuguacc........................   1         0       seq
    ................................................................accaucgaccguugauugu...........................   6         0       seq
    ................................................................accaucgaccguugauugua..........................   3         0       seq
    ................................................................accaucgaccguugauuguac.........................   17        0       seq
    ................................................................accaucgaccguugauuguacc........................   76        0       seq
```
